# Microstructural Characterization and Mechanical Behavior of NiTi Shape Memory Alloys Ultrasonic Joints Using Cu Interlayer

**DOI:** 10.3390/ma11101830

**Published:** 2018-09-26

**Authors:** Wei Zhang, Sansan Ao, Joao Pedro Oliveira, Zhi Zeng, Yifei Huang, Zhen Luo

**Affiliations:** 1 School of Material Science and Engineering, Tianjin University, Tianjin 300072, China; zhang.vv@foxmail.com (W.Z.); ao33@tju.edu.cn (S.A.); yifeihuang@tju.edu.cn (Y.H.); lz_tju@163.com (Z.L.); 2 UNIDEMI, Departamento de Engenharia Mecânica e Industrial, Faculdade de Ciências e Tecnologia, Universidade Nova de Lisboa, 2829-516 Caparica, Portugal; jp.oliveira@campus.fct.unl.pt; 3 School of Mechanical and Electrical Engineering, University of Electronic Science and Technology of China, Chengdu 221116, China

**Keywords:** NiTi shape memory alloys, microstructural characterization, failure behavior, fracture morphology, ultrasonic spot welding

## Abstract

NiTi shape memory alloys (SMAs) are a class of functional materials which can be significantly deformed and recover their original shape via a reversible martensitic phase transformation. Developing effective joining techniques can expand the application of SMAs in the medical and engineering fields. In this study, ultrasonic spot welding (USW), a solid-state joining technique, was used to join NiTi sheets using a Cu interlayer in between the two joining sheets. The influence of USW process on the microstructural characteristics and mechanical behavior of the NiTi joints was investigated. Compared with conventional fusion welding techniques, no intermetallic compounds formed in the joints, which is extreme importance for this particular class of alloys. The joining mechanisms involve a combination of shear plastic deformation, mechanical interlocking and formation of micro-welds. A better bonding interface was obtained with higher welding energy levels, which contributed to a higher tensile load. An interfacial fracture mode occurred and the fracture surfaces exhibited both brittle and ductile-like characteristics with the existence of tear ridges and dimples. The fracture initiated at the weak region of the joint border and then propagated through it, leading to tearing of Cu foil at the fracture interface.

## 1. Introduction

Near equiatomic NiTi is one of the most important shape memory alloys (SMAs) due to its excellent functional properties, namely shape memory effect and superelasticity, combined with high corrosion resistance, as well as, biocompatibility [1,2]. The functional properties originate from a reversible phase transformation between austenite with a B2 cubic structure and a B19’ monoclinic martensite [3,4]. When the material is deformed in the austenitic phase it can exhibit superelastic properties, that is, it can undergo a significant deformation during loading with full recovery to its original shape upon unloading [5]. The excellent mechanical and functional properties exhibited by these alloys make this material widely desired in both medical and engineering fields [6,7,8,9]. To achieve successful fabrication of complex parts of NiTi, it is necessary to develop effective and efficient processing technologies due to the poor machinability of these alloys. In recent years, various welding technologies have been used to join NiTi SMAs both to themselves and to other conventional engineering alloys such as stainless steels [10,11], Ti6Al4V alloys [12,13,14] and Cu-based alloys [15]. Joining techniques such as resistance spot welding [11,16], arc welding [17] and laser welding [10,12] are some examples which are capable to produce defect-free joints. However, a major drawback of the NiTi joints produced by traditional fusion welding techniques is the formation of brittle intermetallic compounds (IMCs) in the weld region, such as Ti_2_Ni, which tend to reduce the mechanical strength of joints [17,18,19,20]. In addition, fusion welding methods can also contribute to significant changes in the transformation temperatures which can impair the potential applications of the joints [19,21].

Considering the possible formation of IMCs in the weld metal, the addition of an interlayer is suggested as a potential solution to adjust the chemical compositions in the weld region and improve the mechanical properties of NiTi joints [12,22]. Cu is a soft metal with a melting point lower than NiTi, and it shows not only high thermal and electrical conductivity, good corrosion resistance and ductility but also a good metallurgical compatibility with NiTi [23,24,25]. For this reason, Cu interlayers have been used in dissimilar laser welding of NiTi to titanium alloys or stainless steel [14,26,27] to limit the mixing of the base material (BM) and increase the mechanical properties of the joints. It has been found that proper selection of the thickness of the Cu interlayer can enhance the mechanical properties of joints by reducing the amount of brittle Ni-Ti-based IMCs [14].

The thermal history experienced by NiTi during welding can significantly affect its shape memory and superelastic properties [28]. Thus, it is necessary to reduce the heat input of the welding process to restrict the thermophysical deterioration in the weld zone [26]. Solid state joining techniques are known for their low heat input and possibility to avoid solidification defects. For example, friction welding has been carried out on NiTi SMAs in recent years [29,30,31]. However, it has been reported for dissimilar joints of NiTi to stainless steel obtained by friction welding that high welding times can promote the formation of brittle phases at the weld interface [30]. Currently, there is a need to develop other solid-state techniques that can successfully join NiTi to itself and to other relevant engineering materials.

Ultrasonic spot welding (USW) is a rapidly developing non-melting joining method which is widely used in plastic forming, electronics and automotive fields [32,33]. As compared to the fusion welding processes, such as resistance and laser welding, USW can produce high strength joints without metal depletion or reduced extension of the heat affected zone. Such translates into almost no detrimental effects produced on the BM. USW is especially suitable for achieving effective joints of miniature components [34,35], such as metallic foils, wires and plates, which make this process particularly interesting to weld materials with lower weldability. Traditionally, studies have been mainly focused on the joining of light materials for weight reduction in industrial products [32,33,34,35]. However, knowledge on the use of USW in NiTi SMAs is currently extremely limited. Thus, carrying out USW on NiTi is a very worthwhile investigation since this technique has great potential for the fabrication of variable electromagnetic switches, radiator fins and other components based on NiTi SMAs.

In the current study, the operating procedures, weld morphology, chemical compositions, interface characteristics and the tensile shear properties of the ultrasonic spot welded NiTi joints with Cu interlayer were analyzed and discussed. Additionally, the effects of welding energy on microstructural characteristics and failure behavior were investigated in detail.

## 2. Materials and Methods

### 2.1. Materials

Ni-Ti shape memory alloy sheets (50.8 at.%), 150 μm thick, were used as the BM. Copper (99.9% purity) foils with a thickness of 20 μm were chosen as the interlayer material. The as-received NiTi alloy sheets were subjected to cold rolling and subsequently stress relieving annealing by heat treatment at 400 °C for 45 min in Ar atmosphere to stabilize the phase transformation temperatures. The DSC result of the BM showed that the transformation start and finish temperatures of austenite and martensite are −45.43, −12.5, 22.91 and −58.75 °C, respectively, which suggests that the NiTi alloy was fully austenitic at room temperature, presenting superelastic behavior. The NiTi sheets were machined into rectangular specimens of 60 mm length and 15 mm width (8 mm width for tensile test of NiTi BM), and the Cu foil was processed into a square shape of 15 mm × 15 mm, which is equal to the overlapped region of the BM specimens. Prior to welding, the oxide layer on the NiTi surface was removed by using a mixed solution of 7.5% HF, 20% HNO_3_ and 72.5% H_2_O for 40–50 s.

### 2.2. Ultrasonic Spot Welding

**Figure 1:**
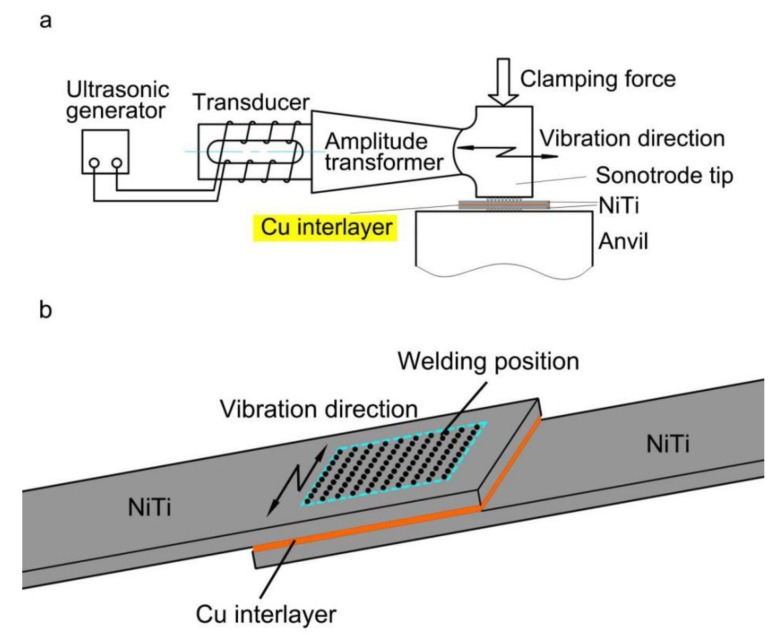
(**a**) Schematic illustration of USW process; (**b**) Schematic diagram showing the welding position of NiTi with Cu interlayer.

USW was performed using a SONICS MSC4000-20. The schematic diagram of the USW system is shown in Figure 1a, which was comprised of ultrasonic generator, transducer, amplitude transformer, sonotrode tip and anvil. The size of the sonotrode tip was a square of 8 mm × 8 mm, consisting of 10 × 10 gridding knurls with a spacing and a depth of 0.8 mm and 0.4 mm, respectively. Figure 1b depicts the relative welding positions of both the NiTi and the Cu interlayer. The ultrasonic vibration direction during the USW process was perpendicular to the longitudinal direction of the NiTi sheets. In this study, the energy control mode was used, during which process the welding time and power input were adjusted automatically. The main process parameters selected were a frequency of 20 kHz, a welding amplitude of 55 μm and a constant clamping pressure of 0.38 MPa, with different energies of 500, 700 and 1000 J, respectively. The USW process was performed at the center of the overlapped position, as shown in Figure 1b.

### 2.3. Microstructural Characterization

The surface profiles of the NiTi welds obtained using different welding parameters were examined using a digital microscope (VHX-2000C, KEYENCE, Osaka, Japan). Additionally, the metallographic observations were performed on the transverse section of the weld, which was parallel to the ultrasonic vibration direction. After mounting the cross sections of the welded joints, mechanical polishing using sandpapers from 240 to 2000 grits, followed by polishing solution agent was performed. The etching process was conducted in an acid solution consisting of 3% HF, 14% HNO_3_ and 82% H_2_O (in volume) with an immersion time of 20–30 s. The microstructures and chemical compositions of the cross sections for different welds were characterized via an optical microscope (OM; GX71, OLYMPUS, Tokyo, Japan) and with a scanning electron microscope (SEM; SU1510, HITACHI, Tokyo, Japan) equipped with an energy dispersive spectroscopy (EDS) analysis system.

### 2.4. Evaluation of Mechanical Properties

Tensile lap shear tests of different joints were performed using an electro-mechanical universal testing machine (AG-100KNA, SHIMADZU, Kyoto, Japan) and the displacement speed was set at 0.1 mm/min. The tensile performance was expressed as joint failure load, determined as the average of three specimens at each welding energy condition. The micro-morphologies and chemical compositions of the fracture surfaces were analyzed by SEM and EDS. In this present work, the trial experiments had shown that in all the welded joint failure always occurred along the bonding interface. Therefore, it was decided to investigate the phase composition of this region using X-ray diffraction (XRD; D8 Advanced, BRUKER, Karlsruhe, Japan) analysis. XRD was performed at 40 kV and 40 mA with Cu-Kα radiation.

## 3. Results and Discussions

### 3.1. Surface Morphology of Welds

**Figure 2:**
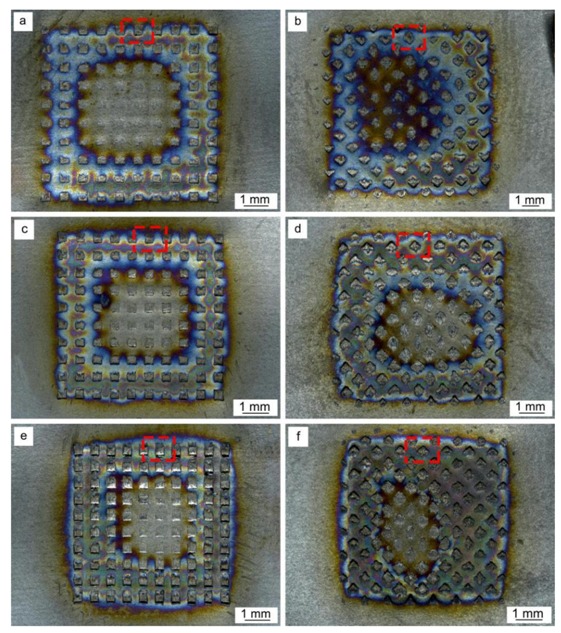
Surface morphology of different samples obtained with different welding energies: (**a**) sonotrode tip and (**b**) anvil sides of 500 J; (**c**) sonotrode tip and (**d**) anvil sides of 700 J; (**e**) sonotrode tip and (**f**) anvil sides of 1000 J. The red squares indicate the regions analyzed to determine the surface indentation depth depicted in Figure 3.

**Figure 3:**
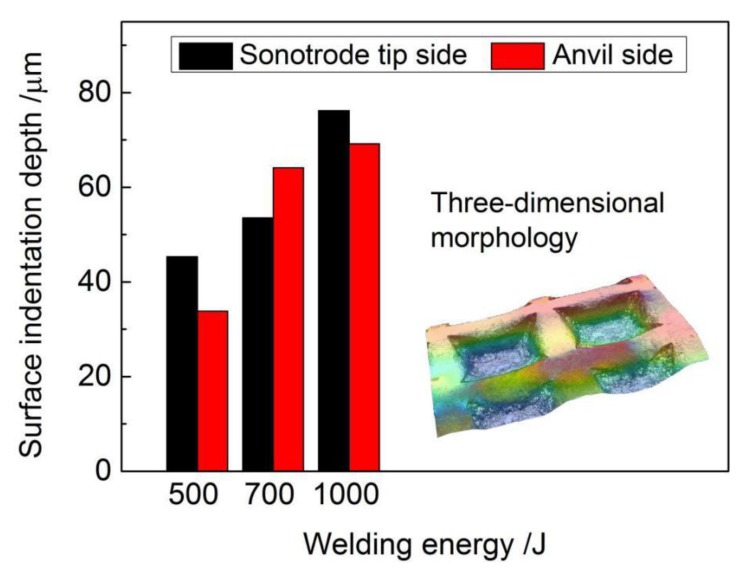
Indentation depths of different samples.

Representative surface morphology at the sonotrode tip and anvil sides of different samples after the USW process are exhibited in Figure 2. Figure 3 shows the depths of surface indentations at the edge of samples as depicted with red rectangles.

From Figure 2, it can be observed that the indentations of sonotrode tip and anvil were distinct on the specimen surfaces, and distinct levels of oxidation were observed on the weld material surface during welding process, due to NiTi’s temperature sensibility and the open structure of the ultrasonic welder. Higher welding energy translates into a higher temperature experienced by the material making it more prone to surface oxidation. With increasing welding energy, the indentation depth in both sides exhibited an increasing trend, as shown in Figure 3, and the insert image shows the three-dimensional morphology of the surface indentation. During the USW process, continuous shear vibrations were applied to the NiTi BM by the action of sonotrode, which quickly generated frictional heat at the weld interface, leading to the rising of temperature and the material was softened, thus shear plastic deformation took place on the material surface [32,36,37,38], resulting in the formation of indentations. Under the same welding parameters, the depth of indentations depends on the accessibility of the sonotrode knurl penetrating into the NiTi material surface, during which a tighter engagement was provided, leading to more relative slippages and friction at the weld interface [36]. Therefore, with the welding energy increasing from 500 J to 1000 J, more frictional heat was generated at the weld interface during the USW process, and then the plastic deformation of NiTi increased since the welding energy was dispersed by shear deformation, resulting in the increasing depth of indentations on the weld material surface aided by a softening behavior of the material caused by the temperature raise.

### 3.2. Bonding Morphology of USW Joints

**Figure 4:**
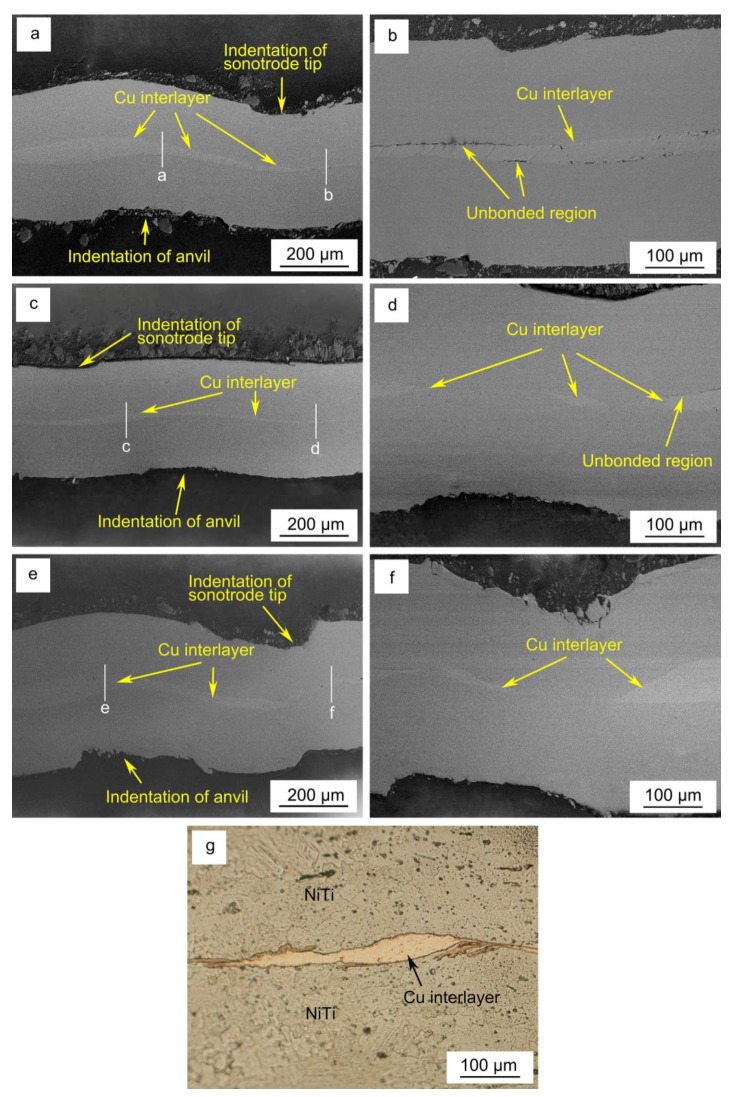
(**a**,**b**) interface morphology of the welded joint obtained at 500 J at the center (**a**) and near the periphery (**b**) of the joint; (**c**,**d**) interface morphology of the welded joint obtained at 700 J at the center (**c**) and near the periphery (**d**) of the joint; (**e**,**f**): interface morphology of the welded joint obtained at 1000 J at the center (**e**) and near the periphery (**f**) of the joint; (**g**) microstructure interface of weld of 1000 J obtained by optical microscope. The white lines in Figure 4. (**a**,**c**,**e**) represent the locals where EDS line scans were performed.

The bonding morphology at the weld interface of the welds obtained by varying the welding energies was examined in the cross section along the center of the joints parallel to the vibration direction as presented in Figure 4.

Figure 4a,b, respectively, depict the interface morphology in the center and near the periphery of the weld obtained for a welding energy of 500 J. From Figure 4a it can be observed that the weld interface showed a wave-like pattern and the thickness of the Cu interlayer was uneven due to the effect of plastic deformation on the material surface, implying the occurrence of material flow at the interface of the deformed material. Under the position of indentation of the sonotrode tip in the material, the thickness of Cu interlayer at the weld interface was thinner than that of the anvil. The weld interface was noticeably intact, resembling a friction-induced bonding characteristic [39]. Joining of NiTi with the Cu interlayer took place by the formation and growth of micro-welds at the weld interfaces owing to the close metal-to-metal contact, which produced mutual diffusion and metallurgical bonding along the interface [33,38,40]. Additionally, unbonded regions were observed near the periphery of the weld, indicating insufficient diffusion during the process. During the USW process, the highest temperature was located at the central area of the weld, thus the bonding between the NiTi and the Cu interlayer was significantly more effective close to this location that in the border of the weld [37]. In comparison, the welded joint obtained with a welding energy of 700 J, which is shown in Figure 4c,d, also presented unbonded gaps along the border of weld interface were also visible. However, these were less often observed due to increased diffusion occurring as a result of the increasing temperatures generated by the increase in the welding energy during the process [39,41].

Observations of the cross-section of the joint obtained at 1000 J showed that both at the center and periphery of the joint complete bonding was obtained, as depicted in Figure 4e,f. It can be observed that the joining interface in Figure 4f was better than that in Figure 4b,d, since no unbonded zones were observed. This can be explained by the greater plastic deformation and increase in temperature that occurred under the indent tips at both the sonotrode tip and anvil sides when 1000 J of energy were used. These results indicate that a good interfacial bonding was obtained during the NiTi with Cu foil USW process.

Figure 4g showed that NiTi and the Cu interlayer were complexly intertwined in the visible weld interface obtained for a welding energy of 1000 J, which contributes to a better bonding along the weld. During the USW process, under the clamping pressure and ultrasonic vibration, the NiTi BM adhered to the Cu foil and then shear deformation, as well as, mutual rubbing of the faying surfaces occurred. Some works [36] have shown that the strength of ultrasonic spot-welded joints is related to interfacial waves, mechanical interlocking and microbonds produced along the weld interface. From the results presented above, it can be concluded that with increased welding energy, more shear friction heat and plastic deformation energy were generated at the weld interface thus promoting joining between the NiTi BM and the Cu interlayer. Mutual extrusion and abrasion between the faying surface of NiTi and the Cu interlayer was a main source for friction heat and plastic deformation. The presence of unbonded zones in the weld interfaces obtained for lower welding energies, would promote premature fracture of joints during tensile testing, since when applying the load, the unbonded areas would become the starting points for crack initiation and propagation until failure of the joint occur.

**Figure 5:**
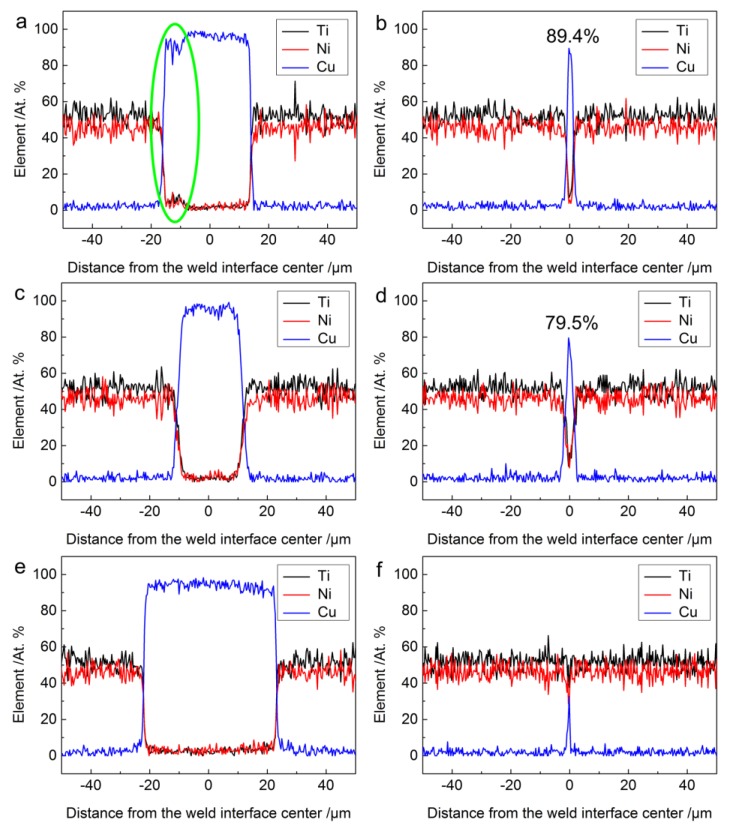
Chemical composition across the weld interface of NiTi joints with Cu interlayer: (**a**,**b**) 500 J; (**c**,**d**) 700 J; (**e**,**f**) 1000 J. The positions where the EDS line scans were performed are indicated by the white lines in Figure 4 (**a**,**c**,**e**).

The EDS line scan analysis at the weld interface center of different joints obtained by various welding energy conditions (the test positions for EDS analysis were depicted with white lines in Figure 4) were conducted to determine the chemical composition and to infer about the potential phases in the interface diffusion layer formed between NiTi and Cu. The results are shown in Figure 5.

It is apparent that the content distribution of Ti and Ni followed the same trend, which is opposite to the change for Cu: a decrease in either the Ni or Ti content would be compensated by an increase of Cu. The EDS line analysis results revealed a smooth and rapid change in composition of both NiTi and Cu across the weld interface border. As indicated by the ellipse in Figure 5a, changes in relative contents of NiTi and Cu elements suggests the onset of slight diffusion at the weld interface. However, the width of the diffusion zone at most weld interfaces was too small to put into evidence the formation of any IMCs layer. Therefore, the joining mechanisms of NiTi with Cu interlayer by USW can be concluded as the combination of solid-state shear plastic deformation, mechanical interlock, the formation and further expansion of micro welds, which was consistent with other previous studies [36,42,43]. Despite the element content fluctuation, it can be seen that the peaks and valleys in the element distribution profiles kept mostly stable in the rich Cu area, as shown in Figure 5a,c,e.

The EDS analysis results also showed that the maximum content of Cu in the thinner Cu area (as depicted in Figure 4) gradually decreased with increasing welding energy, as presented in Figure 5b,d,f, with values of around 89.4%, 79.5% and 32.7%, respectively. It can be reasoned that more Cu foil was squeezed out at some positions of the weld interface with increasing welding energy. Since Cu is a soft metal with high conductivity and low yield strength, it is believed that the existence of Cu layer in the weld can compensate the thermal stress produced during the USW process [44].

### 3.3. Mechanical Performance and Failure Analysis

**Figure 6:**
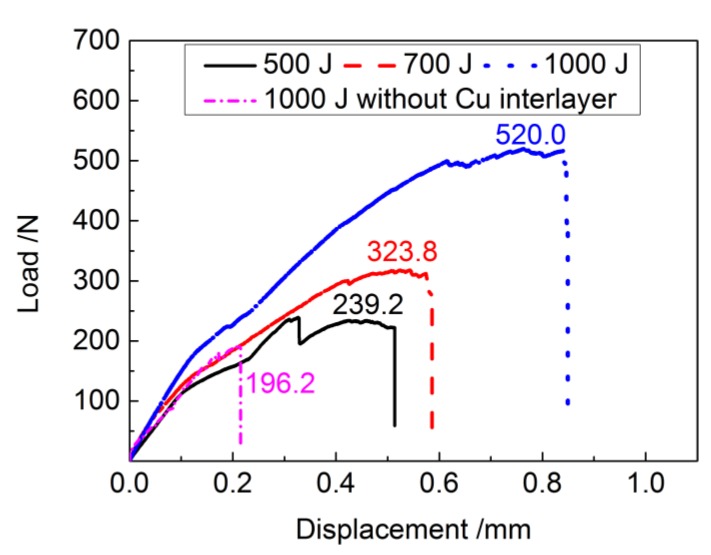
Load-displacement curves of NiTi joints with and without Cu interlayer at different welding energies.

To evaluate the mechanical performance of the ultrasonic spot welded NiTi joints using Cu interlayer, tensile shear tests were conducted. Figure 6 summarizes the load-displacement curves for all welding conditions. Additionally, the load-displacement curve for a NiTi/NiTi weld obtained without Cu interlayer for a 1000 J of energy is also added for comparison.

The failure loads of all joints were defined as the peak tensile load on the load-displacement curve, with each joint representing its own weld strength under certain welding conditions based on the joint performance results. The failure load of the base metal with 0.15 mm thickness and 8 mm width, used in this present work is about 810 N. As shown in Figure 6, it is noticeable that a significantly higher failure load of 520 N was achieved at a welding energy of 1000 J. This higher failure strength is in accordance with the result of weld interface morphology of the 1000 J joint in Figure 4, where good bonding between NiTi and Cu was obtained due to sufficient mechanical interlocking and metallurgical adhesion as a result of a more concentrated vibratory energy. It further indicates that the weld joint with welding energy of 1000 J has the good load capacity, which can be used in distinct engineering fields. Furthermore, the use of the Cu interlayer can improve the mechanical properties of the welded joint: when no Cu interlayer is used the fracture load of the 1000 J weld is of approximately 200 N [45], which is significantly lower than the 520 N obtained when the Cu interlayer was used.

**Figure 7:**
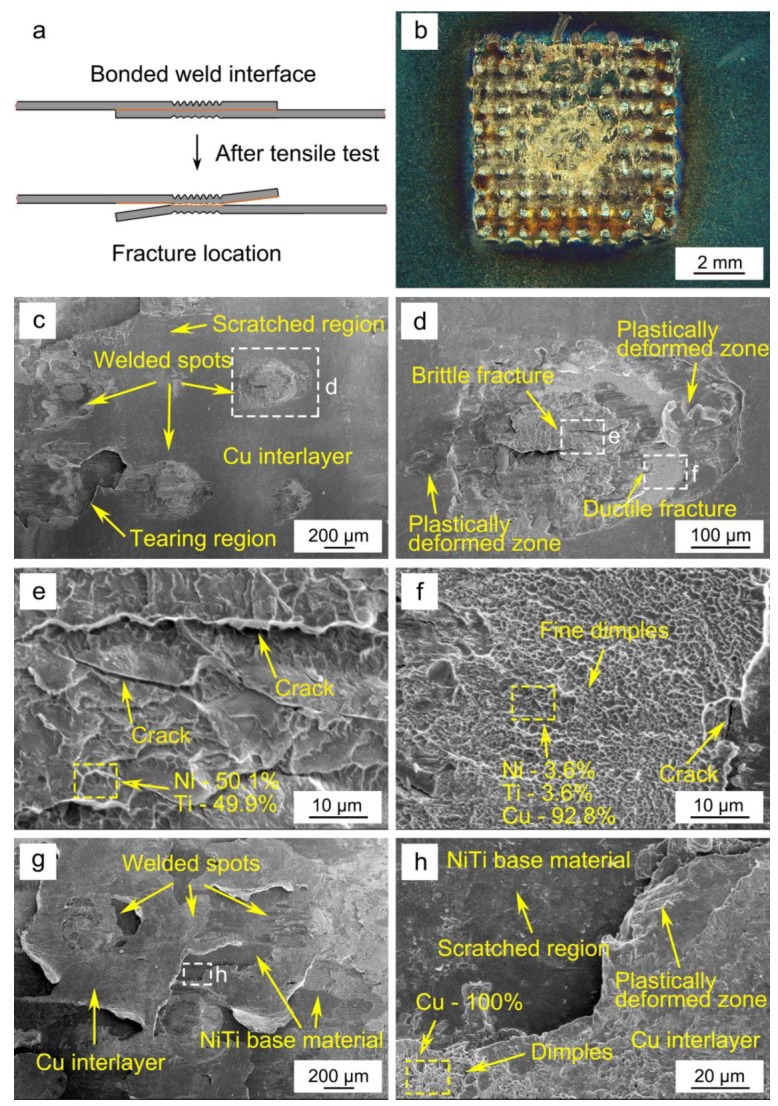
(**a**) Schematic diagram of fracture mode; (**b**–**h**) fracture morphologies of the tensile failed sample made at a welding energy of 1000 J; (**b**) overall view of fracture surface; (**c**) overall view of fracture surface by SEM; (**d**) magnified image of a weld spot depicted in (**c**); (**e**,**f**) magnified image of box in (**d**); (**g**) overall view of Cu interlayer on the fracture surface; (**h**) magnified image of box in (**g**).

In this present work, the fracture location of all the joints occurred at the welded interface. Figure 7a,b show the schematic diagram of the fracture mode and the overall image of the fracture surface of the tensile test sample, respectively, which exhibits the interfacial fracture mode with some welded spots clearly observed. The dominant failure mode was characterized by a tearing behavior along the weld interface: it can be observed that the Cu foils present some tearing features on the fracture surface. In addition, to further understand the weld behavior and failure mode of ultrasonic spot-welded joints, the micro morphology of the corresponding fracture surface of 1000 J joint after tensile shear tests were observed by SEM. The EDS point scan analyses were performed on different areas of fracture surface, with these results presented in Figure 7e,f,h.

The judging criteria for a good weld quality could can be determined by the mechanical properties of the weld but also by the observation of developed weld spots on the fracture surfaces. Three distinct regions were observed on the fracture surface, as exhibited in Figure 7c: weld spots, scratched regions and tearing regions of Cu. The magnified images of the inserts indicated in Figure 7c suggested that on the ultrasonic spot welded NiTi joints with Cu interlayer, more significant deformation was observed under indentations of the sonotrode tip and anvil. As can be seen from Figure 7d, the fracture surface near the periphery of the weld spots was much smother and presented plastically deformed zones, while tear ridges and dimples can be observed at the center, as shown in Figure 7e,f, suggesting that the interface bonding strength near the periphery of weld spot was lower than that at the center of the weld interface.

Brittle fracture characteristics can be observed in the weld spot zone as the high-magnification SEM image presented in Figure 7e reveals. Furthermore, the features in the fracture region of the NiTi weld spot exhibited smooth step patterns and river marks with cleavage-like characteristics. The EDS point analysis showed that this region had a composition of 50.1 at.% Ni and 49.9 at.% Ti, which is good agreement with the expected composition for the as-received NiTi BM. At the weld interface, some intergranular cracking can also be observed due to the strain incompatibility between different grains, which is similar to that observed in NiTi-Cu dissimilar laser welds [15]. In addition, fine dimples also existed in the ductile fracture zone of the weld spot, as shown in Figure 7f. The EDS result shows the composition of dimples in weld spot zone was 3.6 at.% Ni, 3.6 at.% Ti and 92.8 at.% Cu, indicating that ductile failure occurred mainly in the position of softer Cu foil. This can justify the increasing load capacity of the joints obtained with the Cu interlayer: when Cu is used as an interlayer part of the deformation is accommodated by it which provides better mechanical properties than when no interlayer is used.

An overall view of the Cu interlayer on the fracture surface is presented in Figure 7g, and obvious tearing characteristics of Cu can be observed. The magnified image of the insert presented in Figure 7h consisted of both scratched regions and plastically deformed zones. Compared with the NiTi surface, compact dimples existed in the position of Cu interlayer, suggesting that shear fracture occurred through void nucleation, growth and coalescence. During the tensile shear process, a higher tensile load can be transmitted to the 1000 J joint due to a good interface combination, resulting in a higher ultimate tensile load, which is in accordance with the tensile results presented in Figure 6. Additionally, in this study, considering the mechanical performance of joints previously discussed, it is believed that the addition of Cu interlayer into the faying surfaces is critical to enhance the bonding strength due to the increase of friction coefficient [46,47].

The SEM images of the fracture surfaces also verified that the joint was composed of micro welds between NiTi and Cu interlayer. It is possible that the fracture originated from the weak regions in the weld spot border, since this is a suitable location for nucleation and propagation of a crack, and when the crack tip reaches at the boundaries of weld spot, it can easily propagate through it, resulting in the fluctuation of tensile loads due to the existence of the multiple spots connection mechanism of ultrasonic spot-welded NiTi with Cu interlayer, as shown in Figure 6. Meanwhile, the Cu foil between NiTi began to tear, contributing to the process of tensile test until the fracture failure, with the joint being able to deform even more.

**Figure 8:**
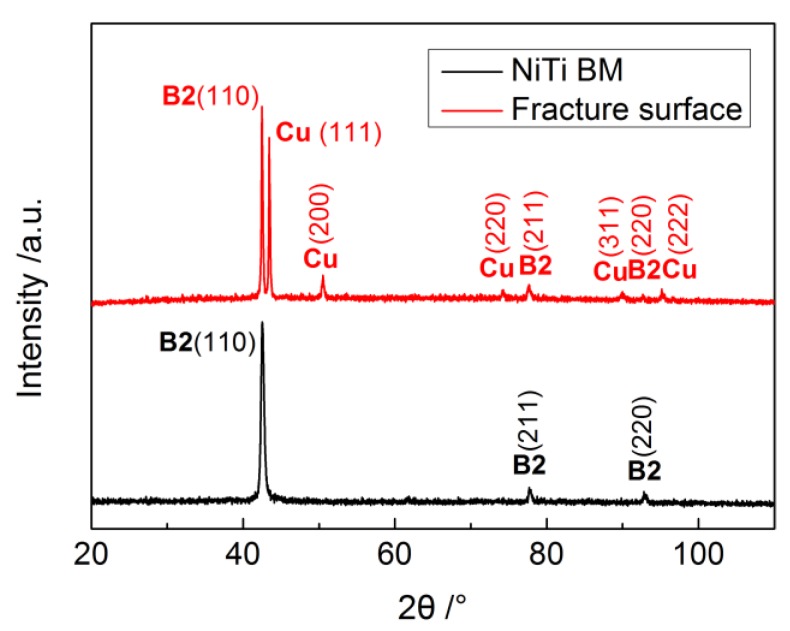
XRD patterns of NiTi BM and fracture surface of 1000 J weld.

X-ray diffraction analysis was carried out at room temperature to evaluate the influence of USW on the phases composition of NiTi weld. The diffraction patterns of both BM and of the fracture surface in the center of the 1000 J weld are depicted in Figure 8.

The indexed pattern of the NiTi BM only consisted of B2 cubic austenite, without traces of B19’ monoclinic martensitic phase, indicating the NiTi BM would be fully austenitic at room temperature. Comparing to the NiTi BM, the fracture surface of ultrasonic spot welded NiTi weld exhibited an additional pure Cu phase due to the addition of the Cu interlayer into the NiTi interface. It is noteworthy that no intermetallic phases, such as Ti_2_Ni or Ni_3_Ti which are usually formed during some fusion welding processes of NiTi [17,18,19,20], were detected in the weld region. Furthermore, the XRD results are consistent with the EDS line scan in Figure 5 where no intermetallic compound layer was found. It is believed that restricting the formation of the brittle phases at the weld interface is beneficial to reduce weld embrittlement, which can further prevent the formation of cold cracking [44]. In addition, the formation Cu-based intermetallics was not observed in this study either, although in fusion-based welding of NiTi to Cu they were already reported [13,15].

## 4. Conclusions

Ultrasonic spot welding of NiTi SMAs with Cu interlayer was performed varying the welding energy levels. The weld morphology, interface characteristics and failure behavior of the joints were analyzed in detail. The following main conclusions can be drawn:

1. With the welding energy increasing from 500 J to 1000 J, more frictional heat was generated at the weld interface and the plastic deformation of NiTi BM increased, leading to the increasing depth of indentations on the weld surface.
2. The ultrasonic spot welded NiTi weld interface presented a wave-like pattern with uneven thickness of Cu interlayer. The occurrence of shear deformation and mutual rubbing of the faying surfaces contributed to the formation and growth of microwelds at the weld interfaces. NiTi and Cu interlayer were intertwined together at the welding energy of 1000 J.
3. EDS line scan and XRD analyses revealed no intermetallic layer formation at the joint interface for all the samples although slight diffusion occurred.
4. The ultimate tensile shear load increased with increasing welding energy due to better mechanical interlocking and metallurgical adhesion at the weld interface.
5. The micro-morphologies of the fracture surface consisted of weld spots, scratched regions and tearing regions of Cu. Both brittle fracture characteristics of NiTi and ductile fracture characteristics of Cu interlayer were observed in the weld spot zone.
